# Polybacterial Periodontal Pathogens Alter Vascular and Gut BH_4_/nNOS/NRF2-Phase II Enzyme Expression

**DOI:** 10.1371/journal.pone.0129885

**Published:** 2015-06-25

**Authors:** Pandu Gangula, Kalpana Ravella, Sasanka Chukkapalli, Mercedes Rivera, Shanthi Srinivasan, Ashley Hale, Keith Channon, Janet Southerland, Lakshmyya Kesavalu

**Affiliations:** 1 Department of Physiology, Meharry Medical College, Nashville, TN, United States of America; 2 School of Dentistry, Meharry Medical College, Nashville, TN, United States of America; 3 Department of Periodontology, College of Dentistry, University of Florida, Gainesville, Florida, United States of America; 4 Department of Medicine, Division of Digestive Diseases, Emory University, Atlanta, Georgia, United States of America; 5 Department of Cardiovascular Medicine, John Radcliffe Hospital, University of Oxford, Oxford, United Kingdom; 6 Department of Oral Biology, College of Dentistry, University of Florida, Gainesville, Florida, United States of America; Temple University School of Medicine, UNITED STATES

## Abstract

Periodontal disease is a highly prevalent chronic inflammatory disease and is associated with complex microbial infection in the subgingival cavity. Recently, American Heart Association supported a century old association between periodontal disease and atherosclerotic vascular disease. We have recently shown that polybacterial periodontal infection led to aortic atherosclerosis and modulation of lipid profiles; however the underlying mechanism(s) has not been yet demonstrated. Altered nitric oxide (NO) synthesis and tetrahydrobiopterin (BH_4_), a cofactor for nitric oxide synthases (NOS) has long been shown to be associated with vascular dysfunction and gastrointestinal motility disorders. We sought to examine the mechanism of periodontal infection leading to altered vascular and gastrointestinal smooth muscle relaxation, focusing on the BH_4_/nNOS pathways. In addition, we also have investigated how the antioxidant system (NRF2-Phase II enzyme expression) in vascular and GI specimens is altered by oral infection. Eight week old male ApoE^null^ mice were either sham-infected or infected orally for 16 weeks with a mixture of major periodontal bacteria *Porphyromonas gingivalis*, *Treponema denticola* and *Tannerella forsythia* to induce experimental periodontitis. Serum, vascular (mesenteric), stomach, and colon specimens were collected at the end of periodontal pathogen infection. Bacterial infection induced significant (p<0.05) reductions in the levels of BH_4_,in ratio of BH_4_:BH_2_+B and also in nitric oxide levels compared to sham-infected controls. In addition, we identified a significant (p<0.05) reduction in eNOS dimerization, nNOS dimerization and protein expression of BH_4_ biosynthesis enzymes; GCH-1, DHFR and NRF2 & Phase II enzymes in infected mice versus controls in both mesenteric artery and colon tissues. However, we found no differences in nNOS/BH_4_ protein expression in stomach tissues of infected and sham-infected mice. This suggests that a polybacterial infection can cause significant changes in the vascular and colonic BH_4_/nNOS/NRF2 pathways which might lead to impaired vascular relaxation and colonic motility.

## Introduction

Periodontal diseases (PD) are among the most common chronic infections of humans, affecting up to an estimated 5–20% of the global population and are associated with a polymicrobial subgingival bio-film. Periodontitis involves progressive loss of connective tissue and the alveolar bone around the teeth, and if left untreated, can lead to the loosening and subsequent loss of teeth [1, 2]. The 'severity' of disease refers to the amount of periodontal ligament fibers that have been lost, termed 'clinical attachment loss' [3]. Periodontitis has been linked to increased local and systemic inflammation and elevated levels of C-reactive protein and interleukin-6 [4, 5]. Individuals with impaired fasting glucose and diabetes mellitus have higher degrees of periodontal inflammation, and often have difficulties while balancing their blood glucose level owing to exposure to the constant systemic inflammatory state, caused by the periodontal inflammation [6–8].

Periodontal disease promotes chronic inflammation due to its polybacterial nature and thus leads to a complex disease cascade which ultimately results in the destruction of the periodontium (alveolar bone, cementum, periodontal ligament, and gingiva). Various studies including ours have shown that periodontal disease is a significant risk factor and contributes to many systemic diseases including atherosclerotic vascular disease (ASVD), stroke, diabetes, rheumatoid arthritis, Alzheimer’s disease, oxidative stress and oral cancer [9–14]. The existence of polymicrobiota in periodontal disease has been established by previous studies [15, 16]. The bacterial species *Porphyromonas gingivalis*, *Treponema denticola* and *Tannerella forsythia*.arestrongly implicated in development of periodontal disease, and together are known as the “red complex” bacteria [15–19].

Periodontal disease is a risk factor for cardiovascular diseases in both diabetic and non-diabetic patients. A strong correlation exists between systemic inflammation and endothelial dysfunction [11, 13]. Recently, the American Heart Association supported an association between PD and atherosclerotic vascular disease (ASVD) [20]. Endothelial dysfunction is one of the initial steps in the events leading to the progression of atherosclerosis and cardiovascular diseases. Reduced NO bioavailability as well as an impaired NO-mediated vasodilation, as a result of endothelial nitric oxide synthase (eNOS) uncoupling and increased reactive oxygen species (ROS), has been known to play a detrimental role in cardiovascular pathologies [21]. Most importantly PD is associated with endothelial dysfunction in patients with coronary artery disease through a decrease in NO bioavailability [22]. Thus, a decrease in NO synthesis may increase the progression of atherosclerosis and CHD in diabetic patients associated with periodontitis [22, 14].

It is well known that NO is an inhibitory neurotransmitter and plays a critical role in the gastrointestinal (GI) tract and its motility. Nitric oxide is synthesized by the activation of neuronal NO synthase (nNOS) in the myenteric plexus. Nitric Oxide that is released from inhibitory neurons (nNOS) plays an important physiological role in various parts of the GI tract [23, 24]. Published data from our laboratory indicate that oxidation of tetrahydrobiopterin (BH_4_; an NOS cofactor with anti-inflammatory and anti-oxidant properties) can lead to oxidative stress, NOS uncoupling and the production of increased inflammatory cytokines including tumor necrosis factor-alpha (TNF-α) [25, 26]. Antioxidants are critical to balance free radicals and protect against oxidative stress. Important to this action is transcription factor NRF2 [nuclear factor (erythroid-derived 2)–like 2], which is encoded by the gene *Nfe2l2* and regulates the expression of Phase II antioxidant and detoxification genes that in turn regulate levels of ROS [25].

Mesenteric arteries, on the other hand, are resistant blood vessels that supply blood to the intestine as well as play a role in the regulation of peripheral blood pressure [27]. It is possible that periodontal pathogens that disseminate hematogenously not only damage vasculature such as mesenteric arteries, but can affect the large intestine and alter motility of the gut, leading to constipation and inflammatory bowel disease. Several clinical, microbiological, molecular, and epidemiological studies have demonstrated that periodontitis is associated with ASVD, but the exact underlying mechanism is elusive. We hypothesize that periodontitis leads to vascular endothelial dysfunction and suppression of antioxidants through impairment of BH_4_/nNOS/NRF2 pathway which leads to elevated vascular inflammation. This in turn might impair gut BH_4_/nNOS/NRF2 pathway, leading to altered colonic motility. In the present study, we demonstrate that there are changes in the BH_4_/nNOS/NRF2 pathway in mesenteric artery and colon samples. Since NO is the principal inhibitory neurotransmitter and controls the peristaltic movements of the gut, we investigated changes in the expression of these pathways in all 3 regions of colon (proximal, mid and distal colon) [24].

## Materials and Methods

### Animal model and oral infection with bacteria mixture

All mice experimental procedures were approved and conducted in accordance with the guidelines of the University of Florida Institutional Animal Care and Use Committee (IACUC, protocol #201304539).

### Ethics Statement

The University of Florida has an Assurance with OLAW (Office of Laboratory Animal Welfare) and follows United States PHS (Public Health Services) policy, the Animal Welfare Act and Animal Welfare Regulations, and the Guide for the Care and Use of Laboratory Animals. The University of Florida is also AAALAC (Association for Assessment and Accreditation of Laboratory Animal Care) accredited. All mice procedures were approved by University of Florida, Institutional Animal Care and Use Committee (IACUC) protocol # 201004539.

*P*. *gingivalis* FDC 381, *T*. *denticola* ATCC 35404 and *T*. *forsythia* ATCC 43037 were used in this study and were cultured anaerobically at 37°C [9–10]. Bacterial concentrations were determined and cells were re-suspended in reduced transport fluid (RTF) at 10^9^ cells/mL. All the 3 bacteria were mixed together in equal quantities and allowed to interact with each other for 5 mins. An equal volume of 4% (w/v) sterile carboxymethylcellulose (CMC; Sigma-Aldrich, St. Louis, MO) (in phosphate buffered saline; PBS) was added to the bacterial mixture and this inoculum was used for oral infection of ApoE^null^ mice. Sham-infected mice were given a 1:1 mixture of reduced transport fluid (RTF) and 4% CMC.

### ApoE^-/-^ mouse oral infection

Our previous studies have established periodontal infection animal model [9] and our published data demonstrated that periodontal infection significantly elevates serum inflammatory biomarkers. These studies have also showed the presence of bacterial genomic DNA in atherosclerosis plaques suggesting that these pathogens disseminate in to the blood and augment atherosclerosis in aorta of atherogenic mice.

Male ApoE^−/−^ mice, eight-weeks-old (The Jackson Laboratories, Bar Harbor, ME) were used in this study. The mice were fed with standard chow and water *ad libitum* and were distributed into two groups (n = 4 mice per group) polybacterial infection and sham-infection. Mice were administered sulfamethoxazole (0.87 mg/mL) and trimethoprim (0.17 mg/mL) in drinking water for 10 days to reduce endogenous microflora. The mouse oral cavity was also rinsed with 0.12% chlorhexidine gluconate. The polybacterial inoculum was administered orally to mice for 4 consecutive days, every alternate week, for a total of 16 weeks under Isoflurane anesthesia. All mice were monitored by the research staff and animal care services (ACS) staff of the University of Florida daily until euthanasia and all the mice appeared healthy throughout the experimental period. Blood, mesentric artery, and gastrointestinal tissues (stomach, colon) were collected for BH_4_/NOS/NRF2-Phase II protein analysis. Superior mesenteric arteries (peripheral resistance arteries, known to regulate blood pressure) were carefully dissected and saved in −80°C for future analysis. Serum was separated and stored at −80°C for BH_4_ and NO measurements.

### Measurement of Biopterin Levels

Biopterin levels were determined in serum obtained from controls and polybacterial-infected mice by HPLC system using electrochemical and fluorescent detection methods as described [21]. Following centrifugation of blood (15 min at 13,000 x *g* at 4°C), the serum samples were transferred to new, cooled micro tubes and precipitated with an equal volume of a solution of cold phosphoric acid (1 M), trichloroacetic acid (2 M), and dithioerythritol (1 mM). The samples were vigorously mixed and centrifuged again for 15 min at 13,000 x *g* at 4°C. The supernatants were injected onto an isocratic HPLC system and quantified using sequential electrochemical (Coulochem III, ESA Inc) and fluorescence (Jasco) detection. HPLC separation was performed using a 250 mm, ACE C-18 column (Hichrom) and mobile phase comprised of sodium acetate (50 mM), citric acid (5 mM), EDTA (48 μM), and dithioerythritol (160 μM) (pH 5.2) (all ultrapure electrochemical HPLC grade) at a flow rate of 1.3 mL/min. Background currents of +500 μA and -50 μA were used for the detection of BH4 on electrochemical cells E1 and E2, respectively. Biopterin and 7,8-BH_2_ were measured using a Jasco FP2020 fluorescence detector [21]. Quantification of BH_4_, BH_2_, and biopterin was done by comparison with authentic external standards and normalized to sample protein content [21].

### Nitric Oxide Levels in Serum

Nitric oxide release experiments were performed as described previously [25]. Animals from both groups (control and infected mice) were sacrificed by CO_2_ asphyxiation and blood was drawn immediately. Niric oxide levels in serum were analyzed as total nitrite (metabolic by-product of NO) according to the manufacturer’s protocol supplied with a commercially available kit (EMD Chemicals, Gibbstown, NJ, USA).

### eNOS, nNOSα Dimerization

Levels of eNOS, nNOSα monomer and dimer were quantified by western blotting via low temperature (LT)-PAGE using vascular, stomach and colon specimens from control and infected mice as described previously [25, 26]. Briefly, LT-SDS-PAGE was performed on ice; 30 μg of protein in standard Laemmli buffer was incubated at 0°C for 30 min and then separated using a 6% separating gel. All gels and buffers were pre-equilibrated to 4°C prior to electrophoresis; the buffer tank was also placed in an ice-bath during electrophoresis to maintain the gel temperature below 15°C. A polyclonal antibody specific to nNOSα (1:1,000 dilution) (Zymed Laboratories, Grand Island, NY, USA), a monoclonal antibody specific to eNOS (1:500) (BD Biosciences, San Jose, CA, USA), anti-rabbit IgG and anti-mouse IgG conjugated with horseradish peroxidase (1:5,000) (Sigma Chemical, St. Louis, MO, USA) were used as the primary and secondary antibodies, respectively.

### Western blot Analysis

The expression of eNOS, nNOSα, BH_4_ biosynthetic enzymes (GCH-1, DHFR) and anti-oxidant enzymes (NRF2, GCSm, GCSc) were quantified in tissue samples using Western blot analysis [22, 26]. Protein concentrations were measured using a Bio-Rad protein assay kit (Bio-Rad, Hercules, CA). Protein samples (30g) were separated by 6%, 15% and 10% SDS-PAGE, respectively. The membrane was immunoblotted with polyclonal/monoclonal primary antibodies (Santacruz Biotechnology, USA) and their respective secondary antibodies. Binding of antibodies to the blots was detected with an enhanced chemiluminescence system ECL (Amersham Pharmacia Biotech, Piscataway, NJ, USA). Stripped blots were re-probed with a β-actin specific polyclonal antibody (Sigma Chemical) to enable normalization of signals between samples. Band intensities were analyzed using Bio-Rad Gel Doc (Bio-Rad, USA).

### Statistics

Data were presented as mean ± standard error (SE). Statistical comparisons between groups was done by using Student's *t*-test or the Tukey test. A *p* value < 0.05 was considered statistically significant.

## Results

### Reduced Serum NO Levels in Polybacterial infected Mice

**Fig 1:**
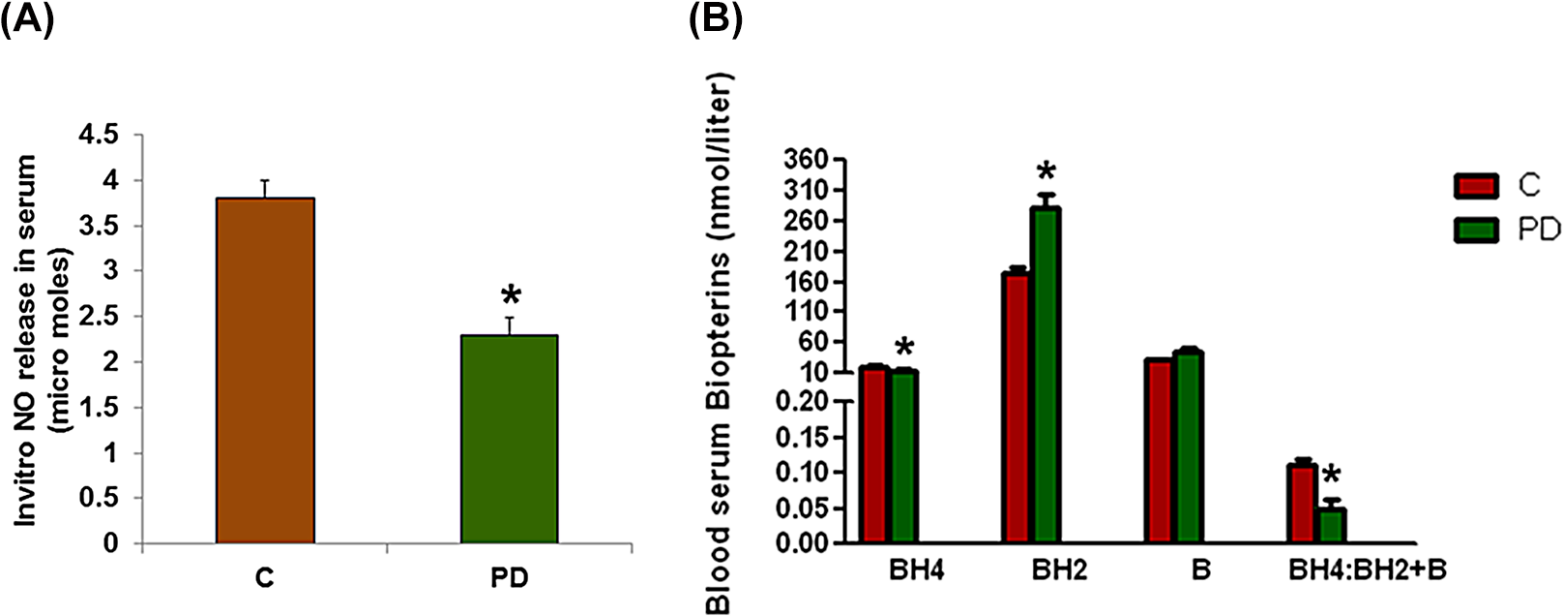
Reduced NO and elevated oxidized biopterin levels in serum from mice with periodontitis versus controls. (A) Oral polybacterial infection significantly reduced serum NO levels (μmoles) and (B) elevated oxidized biopterins levels thus reducing BH_4_ bioavailability when compared to control mice. C = ApoE^null^ male mice; PD = ApoE^null^ male mice with periodontitis (PD). Values are mean ± SE (n = 4 mice per group). Statistical significance was determined by Tukey test. *p < 0.05 compared with controls.

We measured NO levels in serum samples taken from infected and sham-infected mice at sacrifice (at 16 weeks post infection). We found reduced NO levels in serum from ApoE^null^ mice with established periodontitis, in comparison to sham-infected ApoE^null^ mice. This data indicate that polybacterial infection leads to reduced circulating NO levels compared to sham-infected mice (Fig 1A).

### Elevated Serum Oxidized Biopterin Levels in Infected Mice

Fig 1B shows the levels (nmol/L) of total biopterins (BH_4_, BH_2_ and B) in serum from infected and sham-infected mice. A significant reduction was observed in the absolute BH_4_ (reduced form) level as measured by HPLC, whereas there was a significant increase in oxidized BH_2_ levels in infected mice when compared to control mice. The ratio of BH_4_ to total biopterin was therefore significantly (*p* <0.05) reduced in infected mice compared to controls suggesting a lack of BH_4_ bioavailability. Thus, periodontal bacterial infection augmented enhanced oxidized biopterins (reduced BH_4_ availability), and therefore a loss of nNOS function.

### Polybacterial infection Alters Vascular eNOS/nNOS/BH_4_ Pathway

**Fig 2:**
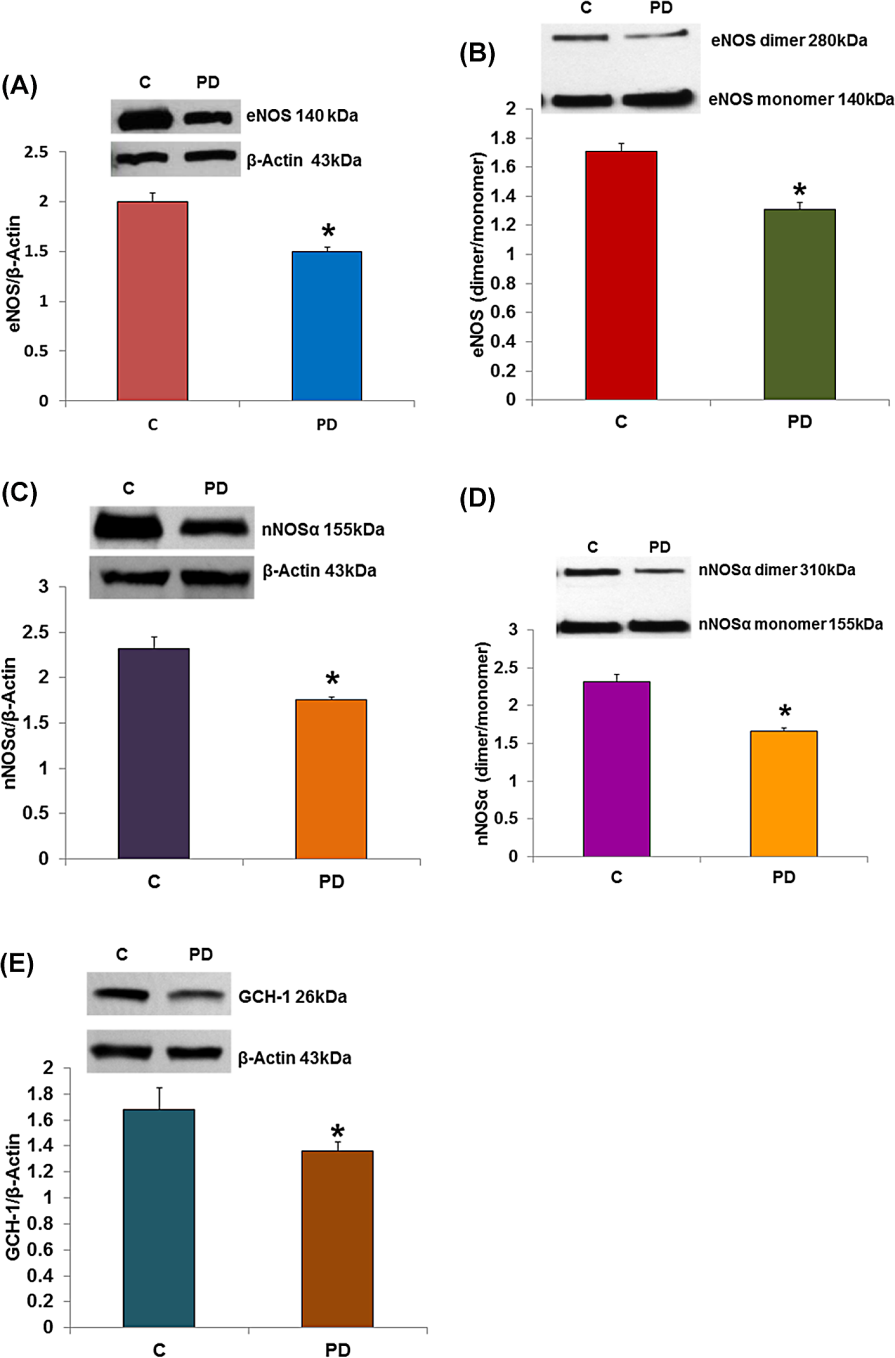
Polybacterial infection alters the vascular BH_4_/eNOS/nNOS pathway. (A) Polybacterial infection reducedeNOS protein expression, (B) dimerization of eNOS, (C) nNOSα protein expressionand (D) dimerization of nNOS and (E) GCH-1 levels, but not (F) DHFR (data not shown) in vascular mesenteric arteries compared to mesenteric arteries of control mice. C = ApoE^null^ males; PD = ApoE^null^ mice with periodontitis (PD). Values are mean ± SE (n = 4). Statistical significance was determined by Tukey test. *p < 0.05 compared with controls.

Since we observed reduced NO levels in the serum of infected mice, we next examined eNOS dimer/monomer expression in mesenteric arteries. Endothelial NOS is a nitric oxide synthase that generates NO in blood vessels and is involved in the regulation of vascular tone by inhibiting smooth muscle contraction. Fig 2A and 2B show a significant reduction in eNOS dimer/monomer (1.71±0.05 vs. 1.31±0.05, *p* < 0.05) and eNOS protein expression (2.0 ± 0.09 vs. 1.5 ± 0.04, *p* < 0.05), respectively in polybacterial infected vascular tissue compared to control mice. Neuronal NOS produces NO in nervous tissue in both the central and peripheral nervous system. Fig 2C and 2D show a significant reduction in nNOS dimer/monomer (2.32±0.09 vs. 1.66±0.04, *p* < 0.05) and nNOS protein expression (2.32 ± 0.13 vs. 1.76 ± 0.03, *p* < 0.05) respectively in infected vascular tissue compared to control mice.

GCH-1 (GTP cyclohydrolase 1) is a rate limiting enzyme responsible for BH_4_ biosynthesis via the de novo pathway. We investigated if the protein expression of GCH-1 was altered in vascular tissues obtained from polybacterial-infected mice. As shown in Fig 2E, there was a significant decrease in GCH-1 protein expression in infected mice vascular tissue compared to control mice (1.68 ± 0.33 vs. 1.36 ± 0.15, *p* < 0.05). We measured protein levels of DHFR (dihydrofolate reducase), an enzyme responsible for the conversion of oxidized BH_2_ into reduced BH_4_ via the salvage pathway. As shown in Fig 2F, there was no significant change in DHFR protein expression was noticed in polybacterial-infected mice vascular tissue compared to control mice (2.03 ± 0.07 vs. 1.98 ± 0.05, *p* < 0.05). The above data suggest that de novo pathway enzymes responsible for BH_4_ biosynthesis were altered by oral polymicrobial infection. This shows that polybacterial infection led to impairment of eNOS/nNOS/BH_4_ pathway in mesenteric arteries. These alterations may lead to reduced vascular function and hypertension.

### Polybacterial Infection Alters Vascular NRF2/Antioxidant Enzyme Expression

**Fig 3:**
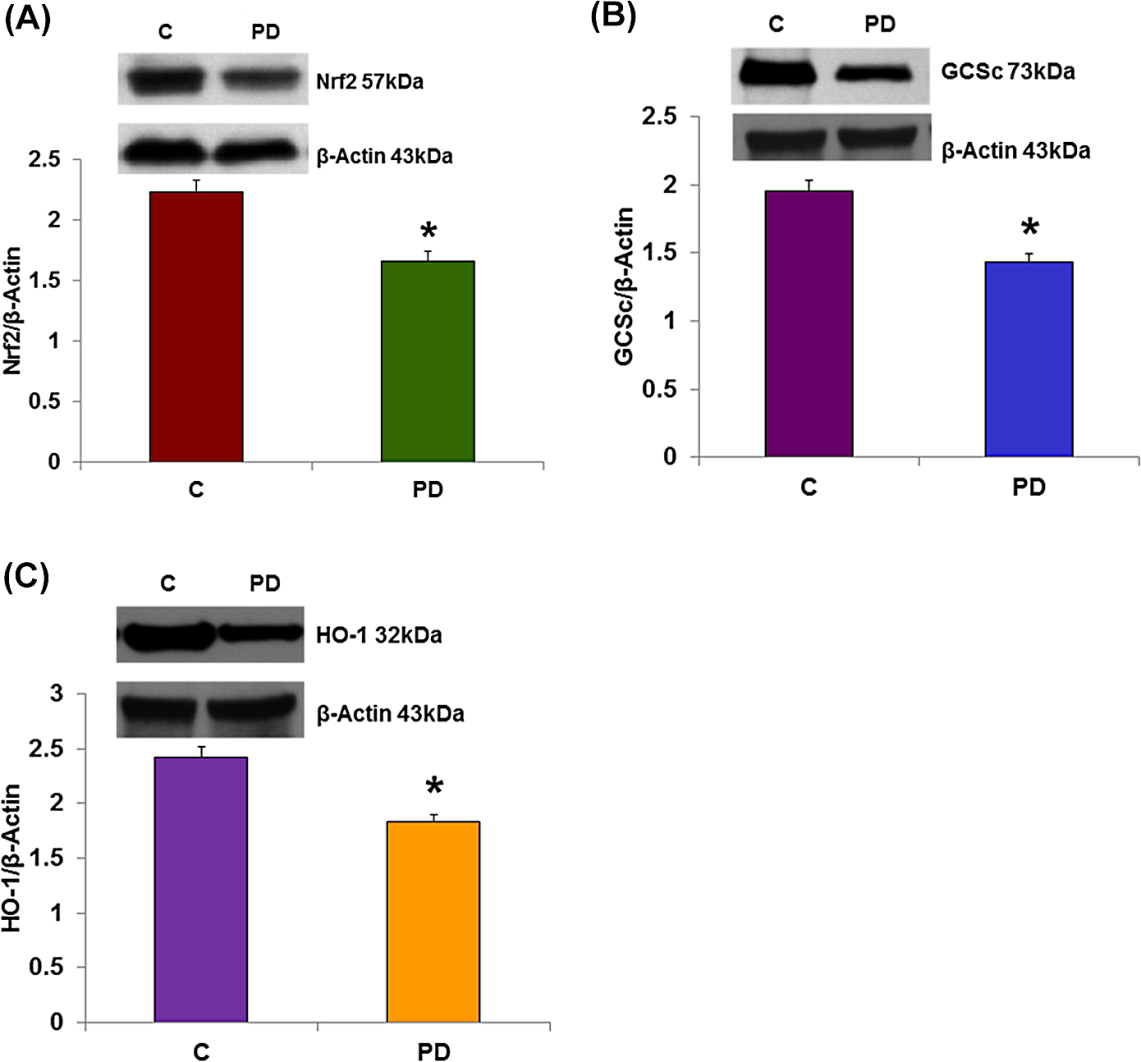
Polybacterial infection reduces vascular NRF2/antioxidant enzymes expression. (A) Polybacterial infection reduced mesenteric vascular protein expression of antioxidant enzymes Nrf2, (B) GCSc and (C) HO-1. GCSm, however, was not affected (data not shown) C = ApoE^null^ males; PD = ApoE^null^ males with periodontitis (PD). Values are mean ± SE (N = 4). Statistical significance was determined by Tukey test. *p < 0.05 compared with controls.

NRF2 (NF-E2-related factor 2) is a transcriptional factor that protects the cells from oxidative stress by activating the antioxidant enzymes including GSH synthesis enzymes (GCSc, GCSm) that are critical for the protection of cells. Heme oxygenase-1 (HO-1) is representative of canonical antioxidant genes that are regulated by NRF2. Fig 3A demonstrates that the protein level of NRF2 was significantly reduced in infected mice vascular tissue (2.23 ± 0.1 vs. 1.66 ± 0.07) when compared to control mice. The protein levels of GCSc and HO-1 are shown in Fig 3B and 3D respectively. The protein levels of GCSc (1.95 ± 0.08 vs. 1.43 ± 0.06) and HO-1 (2.42 ± 0.1 vs. 1.83 ± 0.07) were significantly reduced in infected mice vascular tissue when compared to control mice. However, there was no significant change in the protein expression of GCSm. This novel data suggests that oral pathogen infection induces impairment of vascular NRF2 and antioxidant enzyme expression which may lead to an increase in oxidative stress.

### Impairment of BH_4_/nNOS/ NRF2 Pathway in Proximal Colon Tissue from Orally Infected Mice

**Fig 4:**
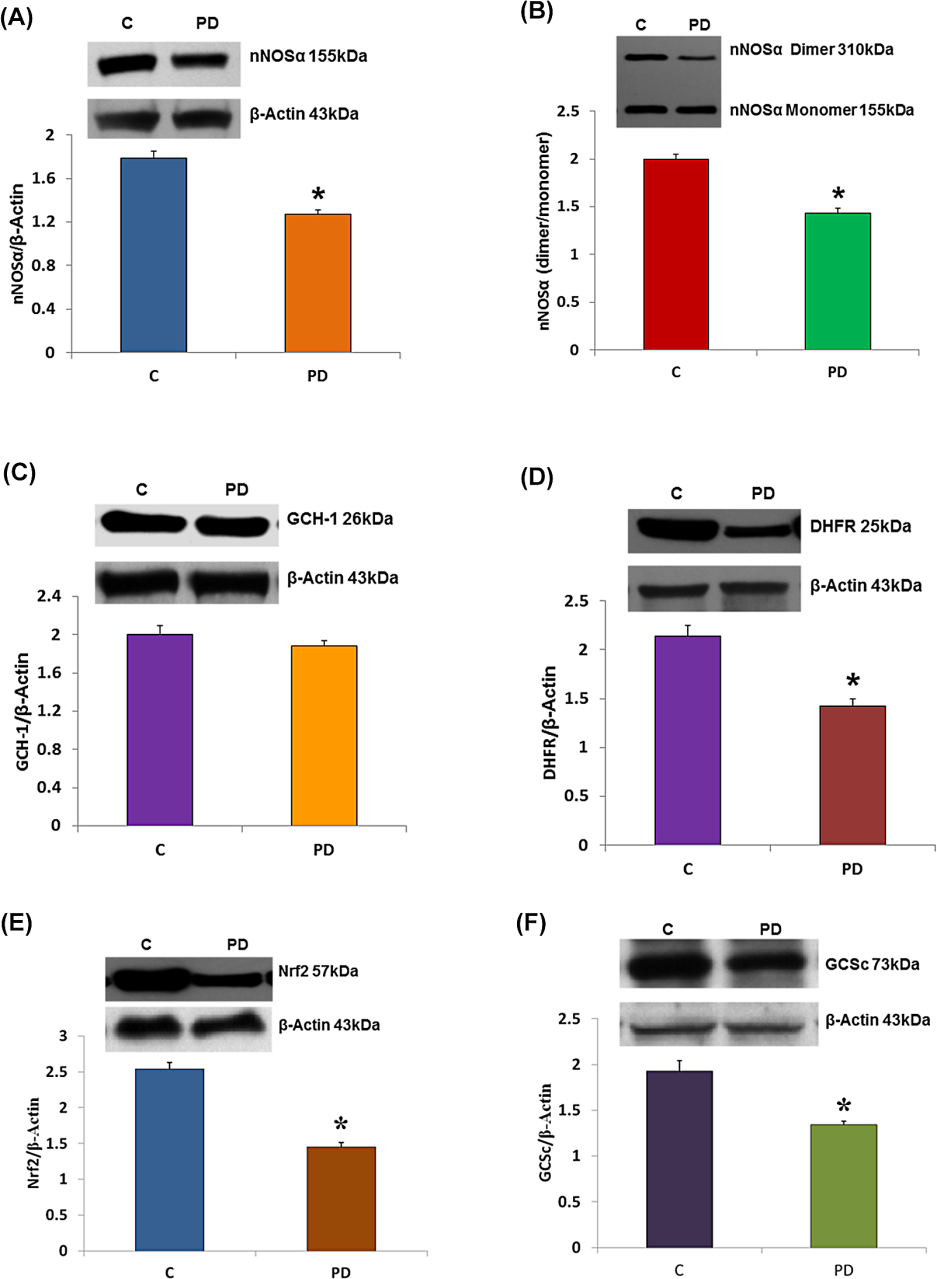
Impairment of BH_4_/nNOS/ NRF2 pathway in proximal colon tissue from infected mice compared to controls. (A) Polybacterial infection reducednNOSα protein expression, (B) dimerization of nNOSα, and (D) DHFR but not (C) GCH-1 expression in proximal colon. Polybacterial infection also reduced antioxidant enzyme protein expression in proximal colon as evidenced by (E) Nrf2 and (F) GCSc compared to controls. GCSm, however, was not affected (data not shown). C = ApoE^null^ males; PD = ApoE^null^ male mice with periodontitis (PD). Values are mean ± SE (N = 4). Statistical significance was determined by Tukey test. *p < 0.05 compared with controls.

The mesenteric artery is the major artery that supplies blood to the large intestine. Since vascular BH_4_/nNOS/NRF2 protein expression is altered in orally infected mice, we then examined if the BH_4_/nNOS/ NRF2 pathway is also impaired in all 3 regions of colon; if so, this could lead to a reduced colonic motility. Fig 4A–4C, 4E and 4F demonstrate that there was a reduced expression of nNOS dimer/monomer (2.0 ± 0.06 vs. 1.43 ± 0.07), nNOS (1.8 ± 0.1 vs. 1.21 ± 0.07) expression, DHFR (2.22 ± 0.09 vs. 1.46 ± 0.05), NRF2 (2.49 ± 0.02 vs. 1.39 ± 0.1) and GCSc (1.87 ± 0.03 vs. 1.23 ± 0.05) in proximal colon of infected mice compared to controls. No significant change was observed in the expression of GCH-1 (Fig 4D) or GCSm (data not shown). To investigate if BH_4_/nNOS/ NRF2 protein expression is altered in other regions of the colon, a series of experiments were conducted in both mid- and distal colon. Our studies indicate that all of these proteins were significantly (p<0.05) altered in both mid- and distal colon as was noticed in proximal colon (data not shown).

The novel data in Fig 4 suggest that oral bacteria that induce periodontitis in these mice lead to impairment of BH_4_/nNOS/ NRF2 pathway which in turn might contribute to colonic motility dysfunctions. However, in assaying the same proteins in stomach from these mice indicated that there were no significant changes in BH_4_/nNOS/NRF2 protein expression suggesting that periodontitis may not have a role in NO-mediated stomach motility (data not shown).

## Discussion

Periodontal disease affects an estimated 116 million Americans and results in complex immune inflammatory lesions. It is a common, chronic inflammatory disease, which has an increased prevalence and severity in patients with type 2 diabetes [28]. Recent report indicates that there is an interrelationship between atherosclerotic vascular disease, diabetes and inflammatory periodontitis [22, 23]. Results from *Sugiyama et al* study indicated that periodontal disease reduces gingival vascular reactivity and decreases reactive hyperemia [29]. Many studies have demonstrated that periodontal bacteria enter the blood stream during mastication, brushing and flossing teeth and during dental prophylactic procedures [9]. Frequent, recurrent transient bacteria have the potential to produce a chronic insult to the vasculature and may contribute to the injury and inflammation that initiates the development of atherosclerosis. In addition, periodontal tissue lesions are recognized as continually renewing reservoirs for the systemic spread of oral bacteria and viruses, and their associated antigens, cytokines and other proinflammatory molecules [30, 31]. Bacterial genomic 16S rDNA from numerous periodontal species, including *P*. *gingivalis*, *T*. *denticola*, *T*. *forsythia*, and *F*. *nucleatum* have been detected in human atherosclerotic plaque lesions [32–35]. Because of interspecies interactions, polybacterial infections have the potential to result in greater deleterious effects as local oral infections and as systemic infection in atherosclerosis vascular system [11]. Furthermore, a recent report indicates that bacteria, viruses, mycoplasma and fungi are associated with ASVD development, demonstrating that the microbiology of the atherosclerotic plaque is complex [12].

While we know that periodontal diseases are polymicrobial and the transition from a healthy subgingival biofilm to a diseased subgingival biofilm occurs as a result of sequential colonization by a broad array of bacteria [36–38], the exact molecular mechanisms that contribute to ASVD are not well understood. It is known that endothelial dysfunction is the initial step in the development of ASVD. Reduced NO bioavailability, as a result of eNOS uncoupling and increased reactive oxygen species (ROS), has been known to play a detrimental role in cardiovascular pathologies [21, 25, 26]. However, the link between NO and PD with regards to systemic vascular function has not been demonstrated. In the present study, we propose that the periodontal bacteria enter the vascular system and alters the BH_4_/NOS (resulting in reduced bioavailability of BH_4_ and NO) and NRF2/antioxidant enzymes pathway (which leads to increased production of ROS and thus increased oxidative stress) in mesenteric arteries which in turn could lead to reduced vasodilation and thereby restricts blood supply to other major organs like colon.”

To date, however, we know little about the oral microbiome and its association with gastrointestinal function, including motility. By far the most heavily colonized organ is the gastrointestinal tract; the colon alone is estimated to contain over 70% of all the microbes in the human body. The human gut has an estimated surface area of a tennis court (300 m^2^) and, as a large organ represents a major surface for microbial colonization [39, 40]. The mesenteric artery is one of the major arteries that supplies blood to the gastrointestinal tract (more specifically the colon). Recently, our laboratory reported that tetrahydrobiopterin (BH_4_), a cofactor for nNOS with anti-inflammatory and antioxidant properties, plays a critical role in gastrointestinal motility [25]. Several other studies in our laboratory showed evidence that BH_4_/nNOS function is impaired in gastric muscular specimens obtained from mouse models of diabetes and chronic estrogen deficiency in ApoE, LDLR (a model of hyperlipidemia), NRF2, and GCSm knockout female mice [26, 41–44]. Since peripheral arteries such as mesenteric blood vessels innervate and maintain blood flow to the gut, we sought to determine if the altered vascular BH_4_ pathway of PD in mice can alter nNOS/NRF2 pathway in stomach and/or colon specimens thus contributing to altered colonic motility and constipation. Hence, we checked the expression of BH_4_ biosynthetic enzymes (GCH-1 and DHFR), nNOS dimer/monomer, nNOS expression, and antioxidant/phase II enzymes (NRF2, GCSc and GCSm) and the BH_4_/ nNOS/ NRF2 pathway was altered in proximal of colon of orally infected mice compared to controls. This data suggests that oral pathogens are able to reach the myenteric plexus located between the smooth muscle layers and alter the nNOS dimerization and NO release to neighboring smooth muscle and regulate the motility of the gut. Next, we checked the BH_4_/nNOS/NRF2 pathway expression in gastric stomach muscular tissues as well, but did not observe any significant changes (data not shown). Collectively, these data suggest that polybacterial infection can cause significant changes in the vascular and colonic BH_4_/nNOS/NRF2 pathway which may lead to altered colonic motility and constipation.

In summary, our findings suggest that a decrease in vascular BH_4_/NO/NRF2 pathway may lead to not only endothelial dysfunction and hypertension, but also to the altered protein expression of members of this pathway in the colon, which may lead to reduced motility and constipation. However, our present studies do not address the fact that periodontal infection decreases vascular relaxation and colonic motility by decreasing NO signaling. In addition, it is not known whether periodontal pathogens directly modulate this pathway and/or indirectly through several other signaling mechanisms, or if it can be influenced by dietary BH_4_. These studies are particularly important and provide value to those subjects [22, 45–49] who have uncontrolled periodontitis and suffer with systemic diseases including diabetes. Nevertheless, our current study for the first time clearly demonstrates and provides strong evidence that a decrease in BH_4_/NO/NRF2 pathway in PD mice could lead to not only endothelial dysfunction and hypertension as seen in the patients [22, 45–49] but also to altered gut function.
